# Digital health interventions for promoting physical activity among adolescents: a systematic review

**DOI:** 10.3389/fpubh.2026.1846042

**Published:** 2026-07-24

**Authors:** Cong Kang, Yuanyuan Luo, Feifei Tian, Huimin Liang, Liqiang Li

**Affiliations:** 1 School of Health and Physical Education, Shangluo University, Shangluo, Shaanxi, China; 2 English Teaching and Research Office, Yishan High School, Fuping County, Weinan, Shaanxi, China; 3 School of Physical Education, Qiannan Normal University for Nationalities, Guizhou, China; 4 School of Physical Education, Xizang Minzu University, Xianyang, Shaanxi, China

**Keywords:** adherence, adolescents, digital health interventions, physical activity, wearables

## Abstract

**Background:**

The issue of insufficient physical activity in adolescents (defined by the World Health Organization as those aged 10–19 years) is a significant worldwide health concern, as 81% do not achieve the suggested levels of physical exercise. Although digital health interventions (DHIs) present promising scalable solutions, their actual performance in practical environments often falls short of anticipated outcomes. This systematic review provides a critical analysis of the existing research on digital strategies aimed at enhancing physical activity among adolescents, emphasizing the factors that contribute to their success, user engagement, and equitable access.

**Methods:**

This systematic synthesis compiles findings from scholarly articles released from 2020 to 2026. The primary outcome was physical activity, measured by daily step counts or minutes of moderate-to-vigorous physical activity (MVPA). Secondary outcomes included engagement, adherence, motivation, psychological factors, equity, and other health-related measures. We included randomized controlled trials, cohort studies, cross-sectional studies, mixed-methods studies, systematic reviews, and meta-analyses. By examining research on a range of digital health technologies—including wearable devices (e.g., fitness trackers, smartwatches), mobile applications, gamified platforms, chatbots, and AI-driven tools—the analysis integrates quantitative data with qualitative insights from co-design studies to provide comprehensive insight into the effectiveness, timing, and target audiences of digital interventions. Methodological quality and risk of bias were assessed using study-design-specific tools: RoB 2 for randomized controlled trials, ROBINS-I for non-randomized interventional studies, AMSTAR 2 for systematic reviews and meta-analyses, NOS for cohort and longitudinal studies, AXIS for cross-sectional studies, CASP for qualitative studies, and MMAT for mixed-methods studies.

**Results:**

The results reveal a persistent disparity between effectiveness and user involvement: while 63% of the examined DHIs demonstrated positive results in their initial assessments, adherence levels dropped from 73% at the start to just 14% by the fifth month. (The 63% = 29/48 studies reporting a significant positive effect on PA. The adherence rates are from a single cohort study tracking device usage over time, not pooled across studies). The success of these interventions is not solely dependent on advanced technology but rather on the integration of three interconnected factors: (1) developmental suitability, where younger adolescents (approximately 10–13 years) are more responsive to playful, sensorimotor activities, whereas older adolescents (approximately 14–19 years) prefer goal-setting and tracking their own progress. (2) relational design, features like team challenges, empathetic AI characters, and customizable avatars can boost confidence by 50% and motivation by 120%; and (3) contextual relevance, data indicates that moderate to vigorous physical activity (MVPA) peaks between 5 and 8 p.m., highlighting the critical difference between screen time (which can hinder activity) and the frequency of physical engagement (which encourages it). Ongoing disparities in equity—such as digital literacy gaps exceeding 35% and reduced engagement among racial and ethnic minorities—emphasize the need for intentional structural and cultural alignment to prevent digital interventions from widening existing inequalities.

**Conclusion:**

Effective digital solutions prioritize the needs of adolescents rather than focusing solely on technology. They emphasize tailored experiences instead of fixed features, foster meaningful connections rather than relying on precise algorithms, and adapt to specific contexts instead of aiming for one-size-fits-all scalability. Future developments should shift away from merely measuring screen time and instead embrace approaches that are rich in experience, affirm individual identities, and promote social justice while incorporating physical activity into the everyday lives of young people.

**Systematic review registration:**

https://www.crd.york.ac.uk/PROSPERO/view/CRD420261415756.

## Introduction

Adolescence, defined by the World Health Organization as the period between 10 and 19 years of age, is a critical developmental stage during which lifestyle habits are formed that often persist into adulthood. Physical activity (PA) plays a fundamental role in healthy adolescent development, supporting cardiometabolic health, bone strength, mental wellbeing, and the prevention of future non-communicable diseases.

Today’s adolescents are more engaged with digital devices than any earlier generation. Smartphones, tablets, smartwatches, and fitness trackers have become ubiquitous, with ownership rates exceeding 80% in many high-income countries and rising rapidly in lower-resource settings. However, the prevalence of physical inactivity is still increasing. Current data indicates that 81% of adolescents worldwide fail to achieve the suggested levels of physical activity (1, 2).

This widespread inactivity leads to serious health risks, including lower cardiometabolic fitness, weakened bone health, higher body fat, and declining mental well-being, particularly affecting youth in underprivileged regions and ethnic minority groups (3, 4). Physical inactivity is also a major modifiable risk factor for noncommunicable diseases, contributing to an estimated 1.6 million deaths annually worldwide (5). Demographic disparities are alarming: only 22% of adolescents adhere to PA recommendations, and just 2.9% of Mexican American girls meet the daily target of 60 min of moderate-to-vigorous physical activity (3).

DHIs—including mobile applications, wearable technology, and web-based platforms—present valuable opportunities to tackle physical inactivity by offering easy access, immediate feedback, and potential for widespread implementation (6–8). These innovations cater specifically to young people, leveraging their comfort with technology and allowing for adaptable participation that bypasses conventional obstacles such as unsafe surroundings, transportation issues, lack of motivation, low self-efficacy, time constraints, and insufficient social support (9, 10). Although around 63% of DHIs demonstrate some success in boosting physical activity among adolescents, the degree of effectiveness varies widely depending on the type of intervention and the context, revealing essential areas needing improvement (2, 11). Nevertheless, existing research highlights notable shortcomings in the effectiveness of these interventions. Comprehensive reviews indicate that many digital solutions do not lead to significant enhancements in physical activity levels or body composition metrics, often due to limitations such as small participant groups, high dropout rates, or inadequate behavior change frameworks (1, 2, 12).

A significant reason for less-than-ideal results is the often poor alignment between intervention design and the developmental, contextual, and relational needs of adolescents (13, 14). Some evidence suggests that young people may prefer digital gaming to physical exercise because of factors such as user-friendliness, adaptability, novelty, and secure, controlled settings (10, 15). However, this finding is based on a limited number of studies and requires further investigation across diverse populations and settings to establish its generalisability. Importantly, while many adolescents use digital technologies (such as fitness apps and social media) outside formal intervention settings, this fact provides the rationale for leveraging these technologies in structured interventions. Digital health interventions themselves frequently overlook the real-life situations faced by underserved adolescents, who deal with safety issues in their neighborhoods and a lack of access to equipment, which diminishes their engagement (4, 9). Additionally, compulsory processes, like the necessity for parents to register their email addresses, may restrict the independence of older adolescents and introduce unwarranted obstacles to access.

Social dynamics also play a crucial role, as adolescents tend to favor collaborative activities over solitary ones and desire community aspects to connect with their peers (16, 17). Digital platforms, particularly social media, can facilitate such social connections, providing adolescents with peer support for physical activity that they may not receive from their families. Research in co-design has shown that adolescents report experiencing negative emotions—such as embarrassment and insecurity—four times more often than positive feelings regarding physical activity, with prevalent obstacles including “lack of confidence,” “fear of failure,” and “lack of motivation” (18, 19).

To achieve effective interventions, it is crucial to focus on highly personalized and developmentally suitable strategies (13, 20). The varying perceptions of technology among different age groups require customized methods: older teens appreciate goal-setting functionalities in wearable devices, while younger individuals often find these devices less useful (15, 21). There is a clear demand from adolescents for culturally relevant personalization, such as options for diverse skin tones and hijabs, along with reward systems that recognize and incentivize sustained engagement (e.g., progress-based badges, personalized feedback for meeting step targets) rather than superficial achievements (e.g., arbitrary points without meaningful reinforcement) (21, 22). Initiatives like the AI-Driven Mechanisms for Ethical Enhancement of Engagement (AIM-EEE) illustrate how interaction data and machine learning can be utilized to adaptively model engagement, refine interventions, and create new content that aligns with the evolving developmental needs of adolescents, all while considering ethical issues like privacy and fairness (13). Research on co-designed solutions supports this methodology; For instance, in one co-design study, chatbots designed with teenage characteristics were associated with a 50% increase in adolescents’ confidence and a 120% increase in motivation, with 73% indicating a strong likelihood of using the service again if avatar customisation was available (19).

Likewise, the Chicas Fuertes program, which combined personalized websites, Fitbit messaging, and social media co-created with Latina teens, led to a remarkable increase in moderate-to-vigorous physical activity from 0 to 64 min per week over six months, surpassing control groups even amid pandemic challenges (3). These results highlight the necessity of centering the voices and experiences of adolescents in digital interventions to address existing challenges and fully leverage their potential for promoting equitable and scalable physical activity.

Despite the significant amount of research on digital interventions aimed at increasing physical activity in adolescents, the current evidence is quite disjointed (23, 24). Quantitative research has primarily examined intervention success, often overlooking the mechanisms at play, while qualitative studies provide insights into personal experiences but struggle to align with quantitative findings (10, 14, 18). Critical questions remain unanswered: What causes the sharp decline in engagement with interventions after initial enthusiasm? How do time-related patterns (e.g., circadian rhythms, timing of use, frequency of interactions) influence outcomes? What role does digital health literacy play in intervention effectiveness and equity? (2, 11).

Given this background, this systematic review aims to:

First, categorize the various forms of digital interventions, such as wearable devices, mobile applications, gamified systems, chatbots, and comprehensive strategies, while assessing their reported efficacy and constraints (1, 2, 25).

Second, identify the factors that motivate and hinder individuals by integrating both numerical and descriptive data regarding the effectiveness-participation divide (10, 11, 19).

Third, investigate the impact of time-related patterns (such as circadian rhythms and the timing or frequency of use) along with contextual elements on the results of interventions (26–29).

Lastly, examine inequalities in digital health literacy, access to devices, cultural adjustments, and the fundamental structural elements involved (4, 22, 30, 31).

## Methods

### Information sources and search strategy

This systematic review was conducted and reported in accordance with the Preferred Reporting Items for Systematic Reviews and Meta-Analyses (PRISMA) 2020 statement (32). The completed PRISMA 2020 checklist is provided as a supplementary file. This review follows a systematic approach, with a search strategy designed to identify key evidence for developing a comprehensive analytical framework. We investigated multiple databases such as EBSCOhost, ISI Web of Science, MEDLINE (PubMed), ScienceDirect, and Scopus. These databases were selected for their comprehensive coverage of peer-reviewed literature in public health, digital health, and exercise science, and for their widespread use in systematic reviews of health interventions.

This specific period was selected to capture the rapid growth of digital health technologies during and after the COVID-19 pandemic, when the adoption of AI-based tools, chatbots, and wearable devices accelerated substantially. For instance, Parker et al. (33) found that 26.5% of adolescents engaged in physical activity on digital platforms during the pandemic, reflecting the increased use of such tools among this age group. This timeframe ensures that the findings are relevant to contemporary practices and technological environments. Additionally, the rapid evolution of technology means that research published as recently as five years ago may assess tools that differ significantly from today’s digital interventions, which could affect the relevance of the findings. Our focus on 2020–2026 balances recency with sufficient breadth to capture meaningful trends.

Throughout the search, we incorporated synonym libraries and free-text keywords associated with terms such as “adolescent physical health”, “digital health interventions”, “artificial intelligence”, “wearable technology”, “health promotion”, “young people”, “and “adolescents”, along with phrases like “digital health”, “digital media”, “digital intervention”, “mHealth”, “mobile health”, “eHealth”, “telemedicine” “and “telehealth.” The search was limited to research articles published between November 2020 and January 2026. Search results were exported to EndNote X20 to remove duplicates, and the subsequent screening process was conducted using Covidence systematic review software. The complete, reproducible search strategy for each database—including Boolean operators, truncation symbols, and database-specific syntax—is provided in Appendix A. This systematic review was registered in PROSPERO (CRD420261415756).

A total of six publications from 2026 were included in this review. Among these, four were classified as ‘Online ahead of print’ (2, 15, 28, 31), one was a fully published article (34), and one was a study protocol (35). The publication status of each 2026 reference is clearly indicated in the reference list using appropriate descriptors (e.g., ‘[Online ahead of print]’ or ‘[Study protocol]’).

### Eligibility criteria

We only included studies that met a few basic requirements. Participants were required to be children or adolescents—that is, aged 8 to 19. Although the review’s title focuses on adolescents (UNICEF defines adolescents as 10–19 years), we included studies with children as young as 8 years to capture developmental transitions and intervention effects across late childhood and early adolescence. This extended range allows us to examine how digital intervention effects may differ across adjacent developmental stages. When applicable, results are disaggregated by age category in the analysis (younger group: 10–13 years; older group: 14–19 years). Children aged 8–9 years are included in the review but not separately analyzed due to the limited number of studies focusing exclusively on this subgroup.

The intervention had to be a digital health tool of some sort. We took a fairly broad view of what counted: wearables, mobile apps, gamified platforms, chatbots, AI-driven tools, and multi-modal digital strategies were all eligible. For comparators, we accepted no intervention, usual care, or a non-digital alternative. In terms of outcomes, we were interested in physical activity—step counts or moderate-to-vigorous activity, for example—but we also considered engagement or adherence, motivation, psychological factors, equity, and any other health-related outcomes that studies happened to report. As for study design, we restricted ourselves to peer-reviewed empirical work published in English between 2020 and 2026. That included randomized controlled trials, cohort studies, cross-sectional studies, mixed-methods studies, and meta-analyses.

### Data synthesis

We decided against a meta-analysis. Given the considerable heterogeneity in study designs, populations, intervention characteristics, and outcome measures across the included studies, a quantitative synthesis would have been methodologically inappropriate (see Cochrane Handbook for Systematic Reviews of Interventions, Chapter 10, on when meta-analysis is not recommended (36)). As the Cochrane Handbook notes, when studies differ substantially in their design, population, intervention, or outcome measurement, pooling effect estimates can produce misleading results, and a narrative synthesis is more appropriate. The studies in this review varied in design, in the populations they sampled, in the digital tools they tested, and in how they measured outcomes. Therefore, we opted for a narrative synthesis instead.

As we worked through the literature, certain outcomes kept surfacing, and we organized our summary around those. One theme was what kinds of digital interventions are out there and whether they actually work. Another was what seems to motivate young people to use these tools—and, just as importantly, what discourages them. We also looked at how physical activity patterns change over time and across different contexts, and at the role of developmental stage and social environment. Finally, we paid attention to where the gaps lie in terms of equity and access.

Given the substantial heterogeneity in study designs, populations, interventions, and outcome measures, a meta-analysis was not appropriate. Instead, we employed a complementary narrative synthesis framework.

First, findings from randomized controlled trials and high-quality quasi-experimental studies (with low-to-moderate risk of bias per RoB 2 and ROBINS-I) served as the primary evidence for claims about intervention effectiveness, such as changes in step counts or minutes of moderate-to-vigorous physical activity.

Second, findings from qualitative, mixed-methods, and co-design studies (assessed with CASP and MMAT) were used to explain the mechanisms underlying intervention success or failure, identify user preferences, and understand barriers and facilitators to engagement.

Third, findings from cross-sectional and cohort studies (assessed with AXIS and NOS) were used to identify factors associated with engagement—such as digital health literacy, neighbourhood safety, and time of day—without claiming causality.

When quantitative and qualitative evidence appeared to conflict, this was explicitly discussed as a source of insight. For example, while randomized controlled trials showed modest effectiveness of wearables on step counts, qualitative studies revealed sharp declines in adherence, highlighting the efficacy-engagement gap that became a central theme of this review.

### Theoretical framework for intervention analysis

To facilitate a structured interpretation of how digital health interventions influence adolescent physical activity, we drew on the COM-B model (Capability, Opportunity, Motivation-Behavior) as an overarching theoretical framework (37). The COM-B model posits that behavior change occurs when there is sufficient capability (psychological and physical), opportunity (social and physical), and motivation (reflective and automatic). This framework was selected because it has been widely used to characterize behavior change interventions and is particularly suitable for understanding the heterogeneous designs of digital tools (e.g., wearables, apps, gamification, chatbots) and their differential effects on engagement and adherence. In our narrative synthesis, we used the COM-B model to: (i) categorize intervention components by the behavioral determinants they primarily target; (ii) explain why multimodal or interaction-enhanced interventions may sustain engagement longer than single-driver interventions; and (iii) interpret the recurring efficacy, engagement gap observed across studies. The application of COM-B was not used to pool effect sizes but to provide a consistent theoretical lens for cross-study comparison and to derive practical design recommendations.

### Risk of bias and methodological quality assessment

To ensure methodological transparency and appropriateness, we applied study-design-specific quality appraisal tools. Randomized controlled trials were assessed using the Cochrane Risk of Bias 2 tool (RoB 2) (38). Non-randomized interventional studies were evaluated with ROBINS-I (39). Systematic reviews and meta-analyses were assessed using AMSTAR 2 (40). Cohort and longitudinal studies were appraised with the Newcastle-Ottawa Scale (NOS) (41). Cross-sectional studies were assessed using the AXIS checklist (42). Qualitative studies were evaluated with the CASP checklist (43). Mixed-methods studies were appraised using the Mixed Methods Appraisal Tool (MMAT) (44). Multiple tools were necessary because no single tool is suitable for assessing all study designs, and this approach follows established guidance from the Cochrane Handbook and other methodological standards.

Two reviewers (CK and YYL) independently assessed each included study. Disagreements were resolved through discussion; if consensus could not be reached, a third reviewer (LQL) was consulted to make the final decision. No study was excluded solely on the basis of its quality appraisal, as we aimed to provide a comprehensive overview of the evidence landscape. However, findings from studies with a lower risk of bias were prioritized in the narrative synthesis. Where evidence was available only from studies with moderate or serious risk of bias, this is explicitly noted in the text to alert readers to interpret those findings with appropriate caution.

A narrative summary of these assessments appears in the results section, and detailed evaluations are presented in the supplementary materials (Supplementary Tables ROBINS-I, AMSTAR 2, NOS, AXIS, CASP, MMAT).

## Results

### Search results

**Figure 1:**
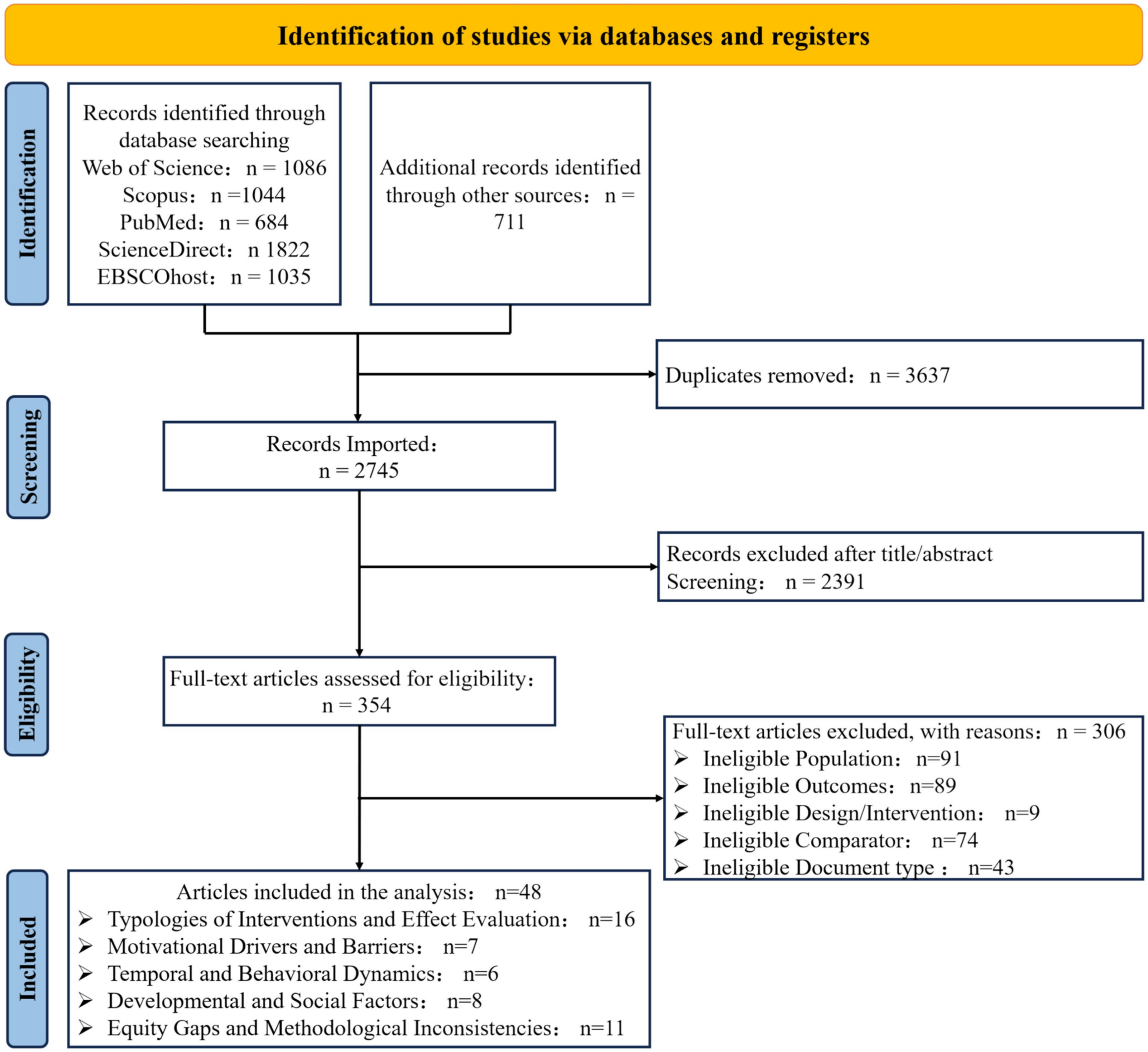
PRISMA 2020 flow diagram of study selection.

The initial search yielded 6,382 records, of which 5,671 were from electronic database searches and 711 from other sources. After removing 3,637 duplicate records, 2,745 records proceeded to the title and abstract screening stage. Following initial screening, 2,391 records were excluded because they were clearly unrelated to the topic of this review or did not meet the inclusion criteria. Subsequently, 354 full-text articles were assessed for eligibility. Of these, 306 articles were excluded for the following reasons: participants did not meet the inclusion criteria (*n* = 91), outcome measures did not meet the inclusion criteria (*n* = 89), study design or intervention type did not meet the inclusion criteria (*n* = 9), comparator conditions did not meet the inclusion criteria (*n* = 74), and article type did not meet the inclusion criteria (*n* = 43). Ultimately, 48 studies met the inclusion criteria and were included in this systematic review. Based on the research topic and core content, the 48 included studies were grouped into five thematic categories: intervention typologies and effectiveness evaluation (*n* = 16), motivational drivers and barriers (*n* = 7), temporal and behavioral dynamics (*n* = 6), developmental and social factors (*n* = 8), and equity gaps and methodological inconsistencies (*n* = 11). Note that individual studies may address multiple themes; the classification reflects the primary focus of each study as judged by the reviewers. The complete literature screening process is presented in Figure 1 (PRISMA 2020 flow diagram).

### Risk of bias and methodological quality

Risk of bias varied across study designs. According to RoB 2, both included RCTs (*n* = 2) had a low risk of bias in randomization, missing data, and outcome measurement, but a moderate risk in blinding (due to the inherent difficulty of blinding participants and outcome assessors in digital intervention research). Non-randomized interventional studies (*n* = 4) generally showed moderate to serious risk of bias per ROBINS-I, primarily due to confounding and selection issues. The Newcastle-Ottawa Scale scores for cohort and longitudinal studies (*n* = 4) ranged from 6 to 7 out of 9 stars, indicating moderate to high quality. Systematic reviews and meta-analyses (*n* = 13) demonstrated moderate quality according to AMSTAR 2, with scores reflecting common limitations including lack of pre-registered protocols and incomplete reporting of excluded studies. AXIS assessments of cross-sectional studies (*n* = 8) indicated moderate quality, with scores ranging from 14 to 16 out of 20, reflecting limitations in sample size justification and non-response handling. Qualitative studies (assessed with CASP) met 7–9 out of 10 criteria on average, and mixed-methods studies (assessed with MMAT) met 4–5 out of 5 core criteria, indicating generally acceptable quality.

No studies were excluded solely on the basis of quality. All were incorporated into the narrative synthesis, although we highlighted their limitations throughout the process. The synthesis emphasizes results from research with minimal bias whenever feasible. In cases where such low-bias evidence was lacking or inadequate, results from studies with moderate bias were included, accompanied by clear warnings. Key conclusions based on individual studies or those with significant bias are clearly marked in the text. Detailed risk-of-bias evaluations for each study can be found in the supplementary materials (Supplementary Tables ROBINS-I, AMSTAR 2, NOS, AXIS, CASP, MMAT). For example, evidence on the effectiveness of wearables (8) had a low risk of bias, whereas evidence on adherence decline (9) had a moderate risk, warranting more cautious interpretation.

### Typologies of digital interventions and their effectiveness

Digital strategies aimed at increasing physical activity among adolescents can be divided into three main categories based on their complexity and functional features (2, 23). The first category, single-driver interventions, accounts for 58% of the research and utilizes a single technology, like smartphones or text messaging, proving particularly effective in encouraging initial behavior changes in the short term (7, 45). The second category, multimodal integrated interventions, makes up 29% of the studies and merges multiple digital tools—such as mobile health applications, wearable devices, and online platforms—making them more effective for the long-term management of at-risk groups, despite their potential complexity, which may affect user adherence (3, 46). Lastly, interaction-enhanced interventions, which constitute 13% of the studies, employ gamification and social engagement to boost emotional and social motivation, effectively promoting short-term behavioral changes through enhanced skills and positive reinforcement (19, 25).

The three categories can be analyzed using the COM-B model, which stands for Capability, Opportunity, and Motivation, a popular framework for understanding behavior change (37). Interventions that focus on a single driver mainly enhance psychological capability, such as teaching individuals how to engage in physical activity, and reflective motivation, like setting goals and providing feedback. In contrast, integrated multimodal interventions tackle several components of the COM-B model at once—improving physical capability via wearable technology, fostering social opportunities through online peer networks, and boosting automatic motivation through tracking progress and sending personalized reminders. Meanwhile, interaction-focused interventions place significant emphasis on social opportunities, such as team challenges and shared objectives, as well as automatic motivation through gamified incentives and supportive chatbot interactions. This theoretical framework clarifies why multimodal and interaction-focused interventions often maintain participant engagement for longer periods compared to single-driver strategies, as they encompass a wider array of factors influencing behavior.

Of the 48 studies analyzed, 29 (63%, calculated as the proportion of studies reporting a significant positive effect on at least one physical activity outcome) indicated positive impacts. The adherence decline from 73% at baseline to 14% at month 5 is from a single cohort study Núñez-Gaunaud and Raya (9) and is not a meta-analytic average. However, this conclusion must be approached carefully, as only 12 of these studies were deemed to have a low risk of bias, while the others exhibited moderate risk stemming from factors like insufficient blinding or significant participant dropout.

Wearable fitness trackers have shown varied impacts on the physical activity levels of adolescents. A comprehensive review and meta-analysis indicated a notable rise in daily steps measured objectively, with a standardized mean difference of 0.37 (8). Nonetheless, the rates of adherence to these devices tend to drop significantly over time: from an initial 73% of users meeting adherence criteria, this figure fell to 67% by the second month (9), 46% by the third, 33% by the fourth, 14% by the fifth, and only 5% by the seventh month (All adherence figures from a single cohort study (9); not pooled across studies.).

Several elements, including a lower starting frequency of physical activity (defined as less than three days per week of moderate to vigorous exercise, associated with an odds ratio of 6.1 for continued usage) and a heightened sense of neighborhood safety, were significant indicators of ongoing device utilization (9). This odds ratio was calculated using a multivariable logistic regression model that factored in initial physical activity levels, perceived safety of the neighborhood, age, and gender as variables (9). Additionally, when combined with customized multi-technology approaches, these trackers led to a significant rise in moderate to vigorous physical activity, with participants in intervention groups increasing their activity from a median of 0 min per week at the start to 64 min per week after six months, in contrast to control groups (3).

The effectiveness of mobile apps and gamified platforms varies based on their design characteristics. When compared to traditional apps, gamified versions lead to slight increases in daily physical activity (approximately 489 additional steps per day) and show some improvements in body measurements, but do not significantly impact blood pressure, lipid levels, blood sugar management, or dietary habits (25). Key design aspects like gamification, personalization, and a focus on user experience play a crucial role in how acceptable these interventions are and their ability to encourage behavior change (14, 21). Students tend to prefer engagement strategies that offer rewards, as earning health points for real-life incentives can help users establish lasting habits through immediate rewards (15, 45).

AI-driven support systems and conversational agents have demonstrated effective outcomes in overcoming psychological obstacles. Phyllis, a conversational agent developed in collaboration with adolescents, achieved a remarkable 50% boost in their confidence regarding physical activity participation (from 53% before the intervention to 80% afterward) and a 120% rise in self-reported motivation (increasing from 33 to 73%) (19). Coaching programs that utilized text messaging to send out weekly standardized messages received high ratings for acceptability (with an average helpfulness score of 5.8 on a 7-point scale) and showed notable enhancements in daily active minutes (an average increase of 20.41 min) and steps taken (an average rise of 924 steps) over a span of 12 weeks (45). Table 1 synthesizes key studies examining the effectiveness of various digital intervention types (wearables, apps, gamification, AI, multi-technology approaches) in promoting adolescent physical activity, providing evidence for the classification framework and effect size estimates discussed.

**Table 1:** Summary of studies on typologies and effectiveness of digital interventions for adolescent physical activity.

Ref. no.	First author	Year	Study population	Core content	Research design	Journal source	Findings/conclusions
Sequí-DomínguezI et al. (1)	Sequí-Domínguez I	2024	Children and adolescents	Evaluates the impact of eHealth interventions on physical activity in children and adolescents	Systematic Review & Meta-Analysis	J Med Internet Res	eHealth interventions have a moderate positive effect; effectiveness is influenced by intervention duration, technology type, and integration of behavior change theories.
Núñez-Gaunaud and Raya (9)	Núñez-Gaunaud A	2025	Underserved adolescents	Relationship between wearable device adherence, physical activity, and safety perceptions in underserved adolescents	Prospective Cohort Study	JMIR Mhealth Uhealth	Adherence dropped from 73% (baseline) to 14% (month 5); low baseline activity (<3 days/week MVPA) and low perceived neighborhood safety were strong predictors of poor adherence.
Carson et al. (3)	Carson J	2025	Latina adolescent girls	Effect of a multi-technology intervention (Chicas Fuertes) on MVPA in Latina adolescent girls	Randomized Controlled Trial	JMIR Mhealth Uhealth	The intervention group increased MVPA from 0 to 64 min/week, with no change in the control group; effects were maintained despite pandemic challenges.
Talens et al. (12)	Talens C	2025	Children and adolescents	Effectiveness of mobile and web-based interventions on healthy eating, obesity prevention, and behavior improvement	Systematic Review(RCTs)	J Med Internet Res	Mobile health interventions can improve dietary behaviors and reduce sedentary time, but have limited effects on objective measures such as BMI; intervention duration and user engagement are key moderators.objective measures like BMI; intervention duration and user engagement are key moderators.
Fan et al. (2)	Fan R	2026	Adolescents	Systematic review of digital health interventions for promoting physical activity in adolescents	Systematic Review	J Med Internet Res	63% of interventions reported positive effects; key success factors include application of behavior change theories, personalized feedback, and integration of social support.
He et al. (7)	He Z	2021	Children and adolescents	Impact of smartphone-based interventions on physical activity in children and adolescents	Systematic Review & Meta-Analysis	JMIR Mhealth Uhealth	Smartphone interventions led to a small increase in daily steps (SMD = 0.37) but had no significant effect on sedentary time; effectiveness is moderated by notification frequency and degree of personalization.
Au et al. (8)	Au WW	2024	Children and adolescents	Effect of wearable activity trackers on physical activity in children and adolescents	Systematic Review & Meta-Analysis	Lancet Digit Health	Wearables significantly increased steps (SMD = 0.37), but adherence declined sharply over time (14% by month 5); effectiveness is related to feedback modality and behavioral support intensity.
Nishi et al. (25)	Nishi S	2024	Adolescents	Impact of gamified digital applications on physical activity and cardiometabolic risk factors	Systematic Review & Meta-Analysis	EClinical Medicine	Gamified apps led to 489 more daily steps compared to non-gamified apps, but had no significant effects on blood pressure or lipids; gamification boosts short-term engagement, but long-term maintenance requires designs fostering intrinsic motivation.
Cummings et al. (45)	Cummings C	2022	Adolescents with overweight or obesity	Impact of a digital health program on physical activity in adolescents with overweight or obesity	Open Trial	JMIR Pediatr Parent	After 12 weeks, daily active minutes increased by 20.41 min, and steps increased by 924; participants showed high acceptance of weekly text messages (mean helpfulness score 5.8/7).
McLaughlin et al. (54)	McLaughlin M	2021	Adolescents	Evaluation of digital support within the PA4E1 school program	Mixed Methods	JMIR Pediatr Parent	Digital support enhanced the implementation of the school program, but technical issues and lack of time limited use; integration into the curriculum and technical training are needed.
McLaughlin et al. (11)	McLaughlin M	2021	Adolescents	Association between engagement with digital health interventions, physical activity, and sedentary behavior	Systematic Review & Meta-Analysis	J Med Internet Res	Engagement (e.g., logins, usage) is positively correlated with intervention effectiveness; self-monitoring features and social functions are associated with higher engagement.
Swindle et al. (56)	Swindle T	2021	Preschoolers	Impact of digital interventions on physical activity in preschoolers	Systematic Review	J Med Internet Res	Limited evidence for effectiveness of digital interventions in preschoolers; most studies focus on parental mediation, but parental engagement is low; suggests developmentally appropriate interactive formats (e.g., animations, simple games).
Shaluhiyah et al. (5)	Shaluhiyah Z	2025	Adolescents	Effectiveness of digital health interventions for preventing non-communicable diseases	Systematic Scoping Review	Dialogues Health	Interventions are effective in promoting healthy diets and physical activity but have limited effects on behaviors like smoking and alcohol use; evidence for long-term effects is insufficient; suggests integrating multiple behavior change strategies.
Chen et al. (46)	Chen X	2025	Adolescents in school settings	Effectiveness of wearable activity trackers on physical activity among adolescents in school-based settings	Systematic Review & Meta-Analysis	BMC Public Health	Wearable activity trackers significantly increased daily steps (SMD = 0.32) and moderate-to-vigorous physical activity (SMD = 0.28) in school-based settings; interventions incorporating goal-setting and feedback components showed larger effects; school-based implementation enhanced accessibility and reduced disparities.
Kalantar et al. (57)	Kalantar H T	2025	Children and adolescents with obesity	Effectiveness of telehealth and wearable device-based interventions for managing childhood and adolescent obesity	Systematic Review & Meta-Analysis	Cureus	Telehealth and wearable device interventions led to modest but significant reductions in BMI (SMD = -0.24) and BMI z-scores; interventions combining both modalities were more effective than single-modality approaches; engagement and parental involvement were key moderators of success.

This table summarizes key references cited in section 3.1 of the review, focusing on the typologies and effectiveness of digital interventions. Core findings are synthesized from the original articles. Ref. No. refers to the original reference number in the paper. MVPA = moderate-to-vigorous physical activity; SMD = standardized mean difference; OR = odds ratio.

The effect sizes presented in Table 1 exhibited significant variation. For instance, devices that monitor activity led to a slight increase in steps (SMD = 0.37, 27), while approaches utilizing multiple technologies resulted in more substantial improvements (64 MVPA minutes per week, 5).

### Motivational drivers and barriers

Adolescents frequently show a greater inclination toward video gaming rather than engaging in physical exercise, influenced by unique motivational elements tied to game design and personal experiences (10). This tendency can be understood through five main themes: features of the game, the environment in which it is played, the results of gameplay, social influences, and availability. Digital games are favored for their design elements such as ease of use, flexibility, and diversity, as well as their engaging attributes like excitement, freedom, and originality. In contrast, physical activities are often viewed as requiring particular gear, time investment, and cooperation with others, which can hinder spontaneous participation (10, 15).

When it comes to motivation, children tend to express a much stronger desire for competition in digital gaming compared to physical activities, largely due to the easy access to online competitive play (15). Conversely, the desire to develop skills is rated more highly in the context of physical activities, indicating that adolescents view the acquisition of practical skills as more achievable through physical movement. This situation illustrates a possible \leisure time conflict,\ where involvement in digital gaming does not indicate a lack of interest in physical activities, but instead highlights different, equally strong motivations influenced by common internal factors like recreation (10, 15).

Engaging in physical activity is hindered by significant obstacles such as concerns about body image, anxiety over being judged, feelings of social isolation, and the belief that obesity makes physical exertion more challenging, leading to both physical discomfort and emotional distress (18). Adolescents frequently indicate that they encounter adverse feelings associated with exercise at a rate that is four times greater than those of positive feelings (18), with common emotional barriers including shame, anxiety, self-doubt, and discomfort (19). Common obstacles they face are insufficient self-esteem, apprehension about failure, and diminished drive.

In contrast, digital platforms utilize fun elements and virtual rewards, which appeal to older teens for setting goals, while younger teens are more inclined toward interactive games that focus on enjoyment and movement without the pressure of tracking performance (20, 21). Table 2 consolidates qualitative and co-design research on adolescents’ psychological motivations, preferences, and barriers regarding digital versus physical activities, informing the motivational framework presented.

**Table 2:** Summary of studies on motivational drivers and barriers to adolescent physical activity in digital contexts.

Ref. no.	First author	Year	Study population	Core content	Research design	Journal source	Core findings/conclusions
Asefi et al. (10)	Asefi A	2024	Adolescents	Reasons for adolescents’ preference for digital games over physical activity games	Qualitative Study (interviews, focus groups)	J Adolesc Health	Five main themes: game features (ease of use, novelty), environmental context (weather-independent), game outcomes (immediate rewards), social interaction (online competition), and accessibility (no equipment needed); physical activity seen as requiring preparation and cooperation.
Krim et al. (18)	Krim F	2025	Obese adolescents	Experiences of obese youth with a therapeutic program using new technologies to encourage physical activity	Qualitative Study	Sci Sports	Participants had positive attitudes toward gamified interventions combining sensors and virtual rewards, but social anxiety and body image remained major barriers; technology alone cannot fully replace interpersonal support.
Moore et al. (19)	Moore RW	2023	Adolescents	Investigating the potential of a conversational agent (Phyllis) to support adolescent health and overcome barriers to physical activity	Co-Design Study	JMIR Form Res	A chatbot co-created with adolescents increased confidence by 50% (53% → 80%) and self-reported motivation by 120% (33% → 73%); 73% of adolescents indicated high likelihood of continued use if avatar customization was available.
Willinger et al. (20)	Willinger L	2024	Children and adolescents	Protocol for evaluating the KIJANI app to promote physical activity in children and adolescents	Mixed Methods Study Protocol	JMIR Res Protoc	The study will combine quantitative (activity tracking) and qualitative (interviews) data to assess the impact of gamification elements (e.g., virtual rewards, adventure tasks) on children’s physical activity and intrinsic motivation; results expected to inform design guidelines.
Wachholz et al. (15)	Wachholz F	2026	Children and their parents	Comparison of motives for digital gaming and physical activity in children, and their parents’ perceptions	Cross-sectional Matched-Pair Study	JMIR Pediatr Parent	Children’s motivation for competition was significantly higher in digital games than in physical activity; motivation for skill development was higher in physical activity; parents often overestimate their child’s interest in physical activity and underestimate gaming addiction risk.
Frazer et al. (21)	Frazer MT	2025	Children aged 8–13	Children’s (aged 8–13) views of a physical activity app	Qualitative Formative Study	JMIR Form Res	Children value personalization (avatars with diverse skin tones, clothing), rewards for effort rather than outcomes, social features (teaming up with friends), and avoidance of public comparisons; they have some awareness of privacy and data security.
D’Halluin et al. (14)	D’Halluin A	2023	Children, adolescents, and parents	Attitudes of children, adolescents, and their parents toward digital health interventions	Scoping Review	J Med Internet Res	Users hold positive attitudes toward digital health tools but are concerned about privacy, data security, and autonomy; parents want to be involved but worry about increased screen time; adolescents prefer fun, social, and non-didactic designs.

This table summarizes key references cited in section 3.2 of the review, focusing on the psychological and contextual factors that motivate or hinder adolescent engagement with digital physical activity tools. Core findings are synthesized from the original articles. Ref. No. refers to the original reference number in the paper.

The qualitative results presented in Table 2 highlight social support and enjoyment as primary motivators, yet they differ regarding particular influences (such as competition compared to avatar customization (15, 21)).

### Temporal and behavioral dynamics

Detailed temporal investigations uncover complex connections between the use of digital tools and the physical activity trends of adolescents (26). One study (26) indicated that the likelihood of participating in moderate to vigorous physical activity (MVPA) peaked during the hours of 5 to 8 p.m. While this conclusion stems from a singular survival analysis and needs further validation, it offers a hopeful foundation for time-specific intervention strategies.

Additionally, disparities focused on equity were noted, with male adolescents having significantly greater odds of meeting MVPA goals during the morning hours (8 a.m. to noon) compared to their female counterparts, and non-school days exhibiting higher activity levels in the same morning period compared to school days (26).

Using applications during the day and evening (from 6:00 a.m. to 7:59 p.m.) is linked to a decline in physical activity, an increase in time spent sitting, and a lower number of daily steps (standardized *β* = −0.21 to −0.07, *p* < 0.001). In contrast, late-night usage (from 10:00 p.m. to 5:59 a.m.) correlates with shorter sleep duration, later sleep and wake times, more sedentary behavior, and fewer steps taken (standardized β = −0.16 to −0.27, *p* < 0.001) (27). Additionally, smartphone activity logged three to twelve hours before going to sleep is a significant predictor of reduced step counts, while no notable relationships are found in the three hours right before sleep begins (27).

The engagement with smartphones, particularly in terms of how long users spend on their screens versus how often they pick up their devices, reveals a two-way relationship. An increase in hourly screen time is associated with a decrease in the number of steps taken, supporting the displacement theory, which posits that more time spent on screens results in reduced physical activity (28). Conversely, a greater number of device interactions appears to be linked to an increase in step counts, suggesting that users might be engaging in physical movement while using their phones (28). This observation from a single sensor-based study implies that brief, contextually relevant interactions with smartphones could complement rather than replace physical activity. Further research across various age demographics and cultural settings is necessary before making definitive conclusions.

When looking at daily patterns, more screen time is associated with a decrease in daily steps (b = −3.08, *p* = 0.025), while the frequency of pickups does not show a significant relationship on a daily basis.

The use of digital technology during weekdays, such as mobile internet access and video gaming, shows a stronger correlation with increased BMI z scores compared to usage on weekends. Specifically, spending more than three hours online each weekday is associated with higher BMI z scores (*β* = 0.30, 95% CI 0–0.61), while extensive weekend internet use does not show a significant link. Likewise, engaging in video gaming for three or more hours on weekdays is related to elevated BMI z scores (β = 0.35, 95% CI 0.07–0.63), with this connection being notably more pronounced for weekday gaming than for weekend activities (*p* = 0.009). Additionally, inadequate sleep plays a role in these relationships, explaining between 8.6 and 17.8% of the effects through indirect means (47).

Engaging in physical activity programs has been proven to enhance sleep quality (SMD = 0.41), shorten the time it takes to fall asleep (SMD = -0.33), and extend overall sleep duration (SMD = 0.28) among children and adolescents. Notably, moderate to vigorous exercise yields more pronounced benefits, highlighting the importance of combining physical activity initiatives with strategies aimed at improving sleep health (34). Table 3 compiles evidence on how the timing of digital device use, screen time patterns, and daily rhythms influence physical activity, sleep, and related health outcomes, supporting the temporal analysis. The temporal data presented in Table 3 reveals significant variability.

**Table 3:** Summary of studies on temporal and behavioral dynamics of digital tool use and physical activity.

Ref. no.	First author	Year	Study population	Core content	Research design	Journal source	Core findings/conclusions
Ortega and Cushing (26)	Ortega A	2020	Adolescents	Developing empirical decision points to improve the timing of adaptive digital health physical activity interventions in youth	Survival Analysis	JMIR Mhealth Uhealth	The probability of achieving average MVPA was highest between 5 and 8 p.m. (OR = 13.19–13.02), suggesting intervention prompts should target this window; proposed the Temporally Augmented Goal Setting (TAGS) strategy.
Alexander et al. (27)	Alexander JD	2025	Adolescents	Daily associations between adolescent smartphone use, sleep, and physical activity using sensor-based measures	Sensor Data Analysis (ABCD Study)	Psychiatry Res	Each additional minute of screen time was associated with a decrease of 6.83 steps; each additional phone pickup was associated with an increase of 13.14 steps; daytime/evening use linked to fewer steps and more sedentary time; late-night use linked to shorter sleep and delayed sleep timing.
Shen et al. (47)	Shen C	2021	Adolescents	Association between digital technology use and BMI	Cross-sectional Analysis (cohort data)	J Med Internet Res	Using the internet or playing video games for >3 h on weekdays was associated with higher BMI z-scores (*β* = 0.30–0.35), but no significant association was found for weekend use; insufficient sleep mediated 8.6–17.8% of the effect.
Burnell et al. (28)	Burnell K	2026	Adolescents	Hourly associations between adolescent smartphone use and physical	activity Hourly Data Analysis (EMA + sensors)	J Adolesc Health	Screen time was negatively associated with steps (−6.83 steps per minute of screen time), but pickup frequency was positively associated with steps (+13.14 steps per pickup); suggests that short, contextualized phone use may accompany activity, while prolonged screen time displaces it.
Ortega and Cushing (29)	Ortega A	2024	Adolescents	Proof-of-concept study of a temporally augmented text messaging bot to improve adolescents’ physical activity and engagement	Proof-of-Concept Study	JMIR Form Res	Personalized text messages delivered between 5–8 p.m. increased adolescent MVPA; the intervention group showed significantly higher activity levels within 30 min post-prompt compared to controls; timing significantly impacts engagement.
Song et al. (34)	Song H	2026	Children and adolescents	Effects of physical activity on sleep in children and adolescents: a systematic review and meta-analysis of randomized controlled trials	Systematic Review & Meta-Analysis of RCTs	European Child & Adolescent Psychiatry	Physical activity interventions significantly improved sleep efficiency (SMD = 0.41), reduced sleep onset latency (SMD = -0.33), and increased total sleep time (SMD = 0.28); moderate-to-vigorous physical activity showed stronger effects; findings support integrating physical activity promotion with sleep health interventions.

This table summarizes key references cited in section 3.3 of the review, which examines how the timing of digital device use and daily patterns influence physical activity and related health outcomes. Core findings are synthesized from the original articles. Ref. No. refers to the original reference number in the paper. MVPA = moderate-to-vigorous physical activity; EMA = ecological momentary assessment.

### Developmental and social factors

Around 26.5% of adolescents indicate they engage in physical activities via digital platforms, mainly through streaming services (40.0%) and online classes (30.2%) (33). Those who use these digital resources are considerably more likely to adhere to physical activity recommendations across various measures (MVPA OR 2.4, 95% CI 1.3–4.3; MSE OR 3.1, 95% CI 2.1–4.4; combined guidelines OR 4.3, 95% CI 2.1–9.0). Notably, 83.5% of these users are female, implying that digital resources may enhance information sharing and self-monitoring, which could particularly boost motivation among girls (33).

Studies have revealed two unique profiles of adolescent engagement. The larger group, termed “family-engaged adolescents” (62.8%), is defined by supportive household dynamics and effective communication norms, along with strong parent–child relationships (16). In contrast, the smaller “At-Risk Adolescents” group (37.2%) is characterized by rigid time restrictions without content guidelines, resulting in lower quality of communication between parents and children. This at-risk demographic shows a notably higher ownership of technology, access to devices in their bedrooms, variations in the timing of smartphone acquisition, and frequent use of social media. They also emphasize the significance of technology in connecting their online and offline lives and fostering social interactions (16, 17).

Motivational routes highlight crucial aspects of development, showing that children can be motivated intrinsically by a desire for self-improvement and personal achievement, or extrinsically by external rewards like social approval, points, and enhancements to their avatars (6, 13). The complexities of social interactions add another layer to engagement, as adolescents often equate online feedback, such as “likes” and comments, with their self-esteem, which can result in an unhealthy fixation on validation. The pressure to uphold online personas through avatars or idealized body representations is seen as a source of stress, leading to negative impacts on mental health (17, 48).

Measures aimed at enhancing digital safety, including tools for parental control and educational materials, have demonstrated a notable enhancement in the monitoring practices of parents (SMD = 0.45). However, there was no significant reduction in the risky behaviors reported by children, highlighting the necessity of integrating these measures with training in effective communication (49).

A model for assessing risk that leverages digital traces, such as time spent online, types of content consumed, and social engagement, can effectively pinpoint adolescents at high risk at an early stage. This model has shown an AUC of 0.82 during external validation, highlighting the promise of digital health approaches for timely intervention (50). Table 4 integrates research on how developmental stage, family dynamics, peer interactions, and cultural context shape adolescents’ responses to digital interventions, providing the evidence base for the developmental and social analysis.

**Table 4:** Summary of studies on developmental and social factors influencing digital intervention outcomes.

Ref. no.	First author	Year	Study population	Core content	Research design	Journal source	Core findings/conclusions
Kim et al. (6)	Kim H	2025	Adolescents, parents, school health teachers	Development of a smart health care service using metaverse and chatbot technologies	User-Centered Design	J Med Internet Res	A co-designed metaverse health service platform, featuring personalized avatars and real-time interaction, significantly enhanced adolescents’ engagement in health management.
Giovanelli et al. (13)	Giovanelli A	2023	Adolescents	Supporting adolescent engagement with AI-driven digital health behavior change interventions	Perspective/Theoretical	J Med Internet Res	Proposed the AI-Driven Mechanisms for Ethical Enhancement of Engagement (AIM-EEE), using machine learning to dynamically model adolescent developmental needs while ensuring privacy, fairness, and algorithmic transparency to promote sustained engagement.
Suh and Yoo (50)	Suh Y	2025	Adolescents	Risk level prediction for problematic internet use from a digital health perspective	Digital Health Perspective (predictive modeling)	Internet Interv	A risk stratification model using digital footprints (e.g., usage duration, content type, social interaction) can identify high-risk adolescents early; the model achieved an AUC of 0.82 in external validation.
Parker et al. (33)	Parker K	2021	Adolescents and adults	Use of digital platforms for adults’ and adolescents’ physical activity during the COVID-19 pandemic	Survey Study	J Med Internet Res	26.5% of adolescents engaged in physical activity via digital platforms, of whom 83.5% were female; adolescents using digital resources were more likely to meet physical activity recommendations (MVPA OR = 2.4, combined guidelines OR = 4.3).
Moreno et al. (16)	Moreno MA	2022	Adolescents	Latent class analysis of digital technology and media use by adolescents	Latent Class Analysis	JMIR Pediatr Parent	Identified two profiles: 62.8% “family-engaged” (parental involvement, rules), 37.2% “at-risk” (time limits only, no content guidance, devices in bedroom); the at-risk group used social media more frequently.
Ziegel et al. (17)	Ziegel L	2025	Adolescents from 11 countries	Perspectives on adolescent mental health and digital communication from 11 countries	Cross-national Survey	J Adolesc Health	Active digital socializing (e.g., private messaging) was associated with lower depressive symptoms, while passive public platform use was associated with anxiety; cultural differences moderated these associations.
Zgambo et al. (49)	Zgambo M	2025	Parents and children	Effect of digital safety interventions on parental practices in safeguarding children’s digital activities	Systematic Review & Meta-Analysis	JMIR Pediatr Parent	Digital safety interventions (e.g., parental control software, educational resources) significantly improved parental monitoring behaviors (SMD = 0.45), but children’s self-reported risky behaviors did not decrease significantly; needs to be combined with communication training.
Goldschmidt et al. (35)	Goldschmidt AB	2025	Adolescents	Study protocol for a user-informed mobile intervention (VIBE) for dysregulated eating and weight gain prevention in adolescents	Study Protocol	Contemp Clin Trials	Describes a user-informed mobile intervention combining ecological momentary intervention and skills training to prevent binge eating and weight gain; will be evaluated using an RCT.

This table summarizes key references cited in section 3.4 of the review, highlighting the role of developmental stage, family, peers, culture, and mental health in shaping adolescents’ responses to digital interventions. Core findings are synthesized from the original articles. Ref. No. refers to the original reference number in the paper. MVPA = moderate-to-vigorous physical activity; AUC = area under the curve; SMD = standardized mean difference.

Research presented in Table 4 highlights the significance of involving families (16) and the development of collaborative chatbots (19). Nonetheless, while digital safety measures enhanced oversight, they did not alter risky behaviors (49), suggesting that social elements interact with context in complex manners.

### Equity gaps and methodological inconsistencies

Variations in digital health literacy create a major obstacle to fair participation. Research shows that many young individuals struggle with digital health literacy, as over 35% of school-aged children in Germany indicate they lack adequate skills in this area. This deficiency is linked to lower involvement in beneficial health practices, such as physical activity. Furthermore, around 50% of German adolescents claim they do not get necessary instruction on digital health literacy within their educational environments (31).

To organize the equity assessment, we utilize the PROGRESS-Plus model, which includes factors such as location, race/ethnicity, job type, gender, faith, education level, economic standing, social networks, as well as age, disability, and sexual orientation (37). This model allows us to differentiate between access equity—covering devices, internet connectivity, and affordability—and engagement equity, which encompasses digital skills, cultural significance, trust, and feelings of safety (4, 22). Focusing solely on access without considering engagement often neglects the needs of marginalized youth. From this analysis, three practical suggestions arise: First, perform assessments to identify specific obstacles; second, collaboratively create content that is culturally appropriate; lastly, offer digital skills training in conjunction with the distribution of devices.

Barriers to access exacerbate inequalities, especially among marginalized adolescent populations identified by race, ethnicity, and economic status. In contrast to their White and higher-income counterparts, Black, Hispanic, or mixed-race adolescents with type 1 diabetes show significantly lower participation in digital health programs. Additionally, low-income youth encounter obstacles to involvement mainly due to access issues, even though they report comparable satisfaction levels once they join. The level of involvement differs significantly across various demographic categories, revealing notable differences between white non-Hispanic adolescents and youth from racial and ethnic minority backgrounds, highlighting persistent shortcomings in the efficacy of digital interventions (4).

In informal settlements in Uganda, displaced young people with limited digital health knowledge faced considerable barriers in obtaining sexual health products and services, influenced by their gender and educational background (30).

Unsurprisingly, digital health inequities look different in Germany, the United States, and Uganda. But three problems cut across all three settings: low digital health literacy, poor device access, and culturally mismatched content. These recurring barriers tell us that although local conditions shape how inequities play out, the case for equity-oriented design is universal (51).

Discrepancies in research methodologies across various studies pose significant obstacles to pinpointing effective strategies (23, 24). A major concern arises from the absence of uniform terminology, as broad terms like eHealth, mobile health, and telehealth cover a wide range of digital health methods, making it difficult to compare outcomes (23). Considerable methodological diversity diminishes the reliability of findings, especially in randomized trials that reveal a high degree of inconsistency (I^2^ = 87.95%) regarding screen time results, while quasi-experimental studies showed no statistical variability (I^2^ = 0%) but yielded highly inconsistent effect sizes (24). Furthermore, insufficient detail in reporting intervention elements worsens these issues, as the descriptions of digital interventions vary greatly, leaving essential questions unresolved about fundamental technological features, non-technical aspects, applications, and target user groups (23, 24).

Digital footprints, including factors like likes, timing of posts, and engagement behaviors, offer a more precise prediction of self-reported well-being compared to individuals’ own accounts of their usage. This underscores both the benefits and ethical dilemmas associated with utilizing \digital footprints\ as an innovative data source for gaining insights into adolescent health (48).

An examination of bibliometric data concerning worldwide research patterns related to digital screen usage and its health implications from 2012 to 2023 has shown significant expansion in this field. Key focus areas include mental well-being, obesity, and sleep issues. While the majority of publications originate from developed nations, there is a noticeable rise in research efforts from developing countries (52).

The d-MUsE Scales, designed for adolescents, have been created and confirmed as a comprehensive tool that assesses various factors such as emotional management, social support, impulsive engagement, and cognitive immersion. This scale is useful for identifying problematic usage and assessing the impact of interventions (17).

Table 5 summarizes studies on disparities in digital health access, literacy, and engagement. The size of these gaps varies by population and context, but the evidence consistently points in the same direction. Methodological differences make cross-study comparison difficult. Randomized trials are highly inconsistent (I^2^ = 87.95%), whereas quasi-experimental studies show no statistical variability (I^2^ = 0%) but still yield highly inconsistent effect sizes (24).

**Table 5:** Summary of studies on equity gaps and methodological inconsistencies in digital physical activity interventions.

Ref. no.	First author	Year	Study population	Core content	Research design	Journal source	Core findings/conclusions
Singh et al. (52)	Singh A	2025	Global research trends (bibliometric)	A bibliometric study on global research trends in digital screen time and associated health factors (2012–2023)	Bibliometric Analysis	Adolesc Psychiatry	Research in this area grew rapidly between 2012 and 2023, with hotspots including mental health, obesity, and sleep; publications are dominated by developed countries, but research in developing countries is increasing.
Sultan et al. (48)	Sultan M	2023	Adolescents	Using social media digital trace data to study adolescent wellbeing	Digital Trace Analysis	Comput Hum Behav Rep	Digital traces (e.g., likes, posting time, interaction patterns) can predict self-reported wellbeing more accurately than self-reported usage; highlights the advantages and ethical challenges of using “digital traces” as a novel data source.
Schröder et al. (31)	Schröder R	2026	Adolescents	Positive evaluation of an online intervention that promotes digital health literacy in adolescents	Randomized Controlled Trial	Comput Hum Behav Rep	The intervention group scored significantly higher on digital health literacy measures than the control group; improved health literacy was associated with subsequent increases in physical activity; supports integrating digital health literacy into school curricula.
Whitehead et al. (4)	Whitehead L	2023	Children and young people	Systematic review of access and engagement with digital health interventions among children and young people	Systematic Review	JMIR Pediatr Parent	Response rates to intervention messages were significantly lower for ethnic minorities (45%) compared to White youth (80%); device access, internet connectivity, and language are key barriers.
Muscat et al. (22)	Muscat DM	2025	Global adolescents	Development of a WHO digital resource (“Your Life, Your Health”) to support universal access to trustworthy health information	Resource Development	JMIR Form Res	A digital resource based on WHO guidelines providing trustworthy health information across multiple topics; disseminated through schools, communities, and online platforms, emphasizing accessibility and cultural appropriateness.
Maass et al. (23)	Maass L	2024	Public health	Mapping digital public health interventions among existing digital technologies and internet-based interventions	Scoping Review	J Med Internet Res	Identified three main types of interventions: monitoring, promotion, and treatment; most studies are from high-income countries; proposes a standardized classification framework to facilitate cross-study comparison and evidence synthesis.
Santis et al. (24)	Santis KKD	2022	Adolescents and adults	Evaluation of digital interventions for physical activity promotion: a scoping review	Scoping Review	JMIR Public Health Surveill	Evaluation methods are diverse, including self-report, sensor data, and system usage logs; there is a lack of uniform outcome indicators, suggesting the use of core outcome sets; mixed methods designs help understand intervention mechanisms.
Cascio et al. (53)	Cascio CN	2023	Adolescents	Protocol for a multimethod longitudinal study on the effect of technology and digital media use on adolescent health and development	Multimethod Longitudinal Study Protocol	JMIR Res Protoc	Plans to combine sensor data, experience sampling, and interviews to track the dynamic relationships between digital media use, physical/mental health, and social development; emphasizes an ecological perspective.
Okumu et al. (30)	Okumu M	2025	Displaced youth (Uganda)	Digital health literacy and its role in awareness of and access to sexual health products and services among displaced youth in Uganda’s informal urban settlements	Community-based Cross-sectional Study	J Med Internet Res	Among displaced youth in Ugandan informal settlements, those with lower digital health literacy had significantly fewer opportunities to access sexual health products and services; gender and education level were moderating factors.
Antons et al. (55)	Antons S	2025	Adolescents	Conceptualization and validation of the Digital Media Use Effects Scales (d-MUsE Scales) for adolescents	Scale Development	Comput Hum Behav Rep	Developed and validated a multi-dimensional scale (d-MUsE) measuring constructs like emotion regulation, social compensation, impulsive use, and cognitive absorption; can be used for screening problematic use and evaluating intervention effects.
Hematabadi et al. (51)	Hematabadi et al.	2025	Young adults	Social media-based physical activity interventions for obesity prevention	Narrative review	Youth	Highlights equity barriers including digital divide, cultural mismatch, and accessibility issues; emphasizes intersectional and culturally responsive design in digital health promotion.

This table summarizes key references cited in section 3.5 of the review, addressing disparities in digital health access, literacy, and engagement, as well as methodological challenges in the field. Core findings are synthesized from the original articles. Ref. No. refers to the original reference number in the paper.

## Discussion

### Summary of key findings

This review compiles findings from various research methodologies to clarify the complex realm of digital strategies aimed at enhancing physical activity among adolescents (1–31, 33–35, 45–50, 52–57). The results suggest a key understanding: the effectiveness of these interventions may depend more on how well they align with the developmental requirements, social preferences, and real-life contexts of adolescents, rather than solely on the advanced nature of the technology used (2, 13, 14). Around 63% of these digital strategies show beneficial impacts on physical activity levels; however, the field is marked by a significant gap between efficacy and engagement: participation rates typically drop from 73% at the start to just 14% by the fifth month (descriptive trends from included studies, not pooled estimates), suggesting that initial enthusiasm does not translate into sustained behavioral change (2, 9, 11).

Evidence typology: (i) Empirical (RCTs/quasi-experimental): effectiveness claims. (ii) Mechanistic (qualitative/co-design):user preferences/barriers. (iii) Exploratory (single studies/cross-sectional/protocols): hypothesis-generating.

### Comparison with prior reviews

Numerous earlier systematic reviews have investigated digital strategies aimed at enhancing physical activity among adolescents, primarily concentrating on their effectiveness (1, 2, 7, 8, 25, 46, 56, 57). In contrast, this review uniquely incorporates three key aspects—developmental appropriateness, relational design, and contextual significance—across a wider variety of intervention formats (such as wearables, mobile applications, gamified systems, chatbots, and AI-based tools) and research methodologies (including quantitative, qualitative, and mixed-methods approaches). Instead of merely inquiring \what is effective\we explore\what is effective, for whom, in what circumstances, and why. This comprehensive, person-centered synthesis builds on previous research by converting effectiveness insights into practical design guidelines for both researchers and practitioners, thereby advancing from a mere catalog of successful interventions to a deeper understanding of the factors that contribute to the success or failure of various interventions.

### The efficacy- engagement gap: interpretation and implications

The significant drop in compliance over time poses a major obstacle that goes beyond specific types of interventions (8, 9). This trend is not exclusive to digital methods; rather, it is intensified by the initial excitement surrounding technology (2). While curiosity may spark initial involvement, long-term commitment necessitates a stronger foundation of motivation (11). Empirical randomised controlled trial evidence shows that participation declines when initiatives fail to transition from external incentives to internal drivers (13, 15, 25). Sustained involvement often shares common traits identified in qualitative and co-design studies, including gradual challenges, a sense of social duty, personalization, and an emphasis on personal development instead of comparing oneself to others (6, 19, 21).

The variation in effect sizes observed across different intervention types (e.g., wearable trackers vs. multi-technology approaches) suggests that both the intensity of the intervention and the surrounding context are as crucial as the technology itself.

Additionally, the difference between the amount of time spent on screens and the frequency of interactions adds complexity to the engagement discussion (28). Although spending more time in front of screens typically leads to less physical exercise, engaging more frequently in social interactions tends to result in higher step counts. This implies that short, contextually relevant engagements may actually promote physical activity rather than impede it. Such complex dynamics are often overlooked in broad screen time analyses, underscoring the necessity for more detailed measurement techniques (48, 55).

### Developmental and relational dimensions of engagement

The results highlight that developmental suitability is fluid rather than fixed (13, 21). Evidence from qualitative and cross-sectional studies suggests that younger teens, typically aged 10 to 13, may engage more enthusiastically in playful activities that are rich in sensory experiences and focus on immediate enjoyment. In contrast, older teens, aged 14 to 19, appear more inclined toward activities that emphasize goal achievement, tailored feedback, and self-monitoring elements that promote independence and personal identity (15, 20, 21, 45). Programs that do not adapt to these developmental changes may lead to quick disengagement, as younger adolescents often struggle to engage with connected devices, while older adolescents perceive them as beneficial (8, 9).

The structure of relationships is just as crucial as the functional aspects (14, 19). Research consistently challenges the notion of individualistic engagement approaches. Young people do not view digital tools merely as standalone instruments for behavior change; instead, they anticipate these tools to facilitate social interactions, shared experiences, and collective understanding. Collaborative challenges, intimate peer groups, co-created conversational agents that balance authority and warmth, along with options for personalizing avatars, act as relational supports that enhance perceived significance and lessen psychological risks (6, 19, 21). Similar qualitative observations from co-design studies highlight the value of relational features (19).

Adolescents clearly express a need for culturally sensitive customization, such as varied skin tones and hijabs, as well as reward systems that reflect effort rather than superficial criteria (21, 22).

### Temporal and contextual considerations

The timing of events influences the chances for effective interventions in ways that have not been fully explored in existing research. Exploratory survival analysis evidence from a single study suggests that the probability of participating in moderate to vigorous physical activity (MVPA) may peak between 5 and 8 p.m., suggesting a potential strategy for interventions tailored to specific times (26, 29). This method aligns with contemporary frameworks for adaptive interventions delivered at the right moment, as well as the Temporally Augmented Goal Setting (TAGS) approach.

Adolescents’ capacity to convert digital health information into active lifestyles is significantly hindered by external factors that are often beyond their control (4, 9). Issues such as safety in their neighborhoods, availability of equipment, transportation challenges, and school timetables exert a greater influence on their physical activity than any single intervention aspect can achieve (9, 33). Those from underserved backgrounds encounter safety issues and lack of equipment that hinder their participation, while obligatory processes, like requiring parental email registration, limit the independence of older youth and create obstacles to access (4, 6).

Engaging in physical activities has been proven to greatly enhance sleep quality among children and adolescents. This indicates that combining initiatives that encourage physical exercise with those aimed at improving sleep health may provide complementary advantages for the overall wellbeing of adolescents (34).

### Equity and implementation challenges

Ongoing disparities in equity require immediate action (4, 30). In certain adolescent groups, over 35% struggle with digital health literacy (31), while racial and ethnic minority youth face lower rates of enrollment and participation (4). Additionally, obstacles such as limited access to devices and broadband connectivity (22) hinder the potential of digital interventions to serve as widespread solutions. Importantly, metrics related to \screen time\ fail to highlight these disparities—they measure usage volume rather than the nature of engagement, focusing on passive viewing instead of active participation (48, 55).

Qualitative and mixed-methods research indicates that an equity-focused design approach requires active collaboration with underrepresented youth (30, 53), the incorporation of culturally relevant elements (21, 48), and structural changes to overcome access challenges (22, 30).

Additionally, it highlights the importance of ethical AI to guarantee fairness in algorithms and safeguard privacy (13). Data from a systematic review of access and engagement (4) show that 80% of intervention messages were effective among white non-Hispanic youth, compared with just 45% among racial and ethnic minority groups, emphasizing that without a conscious effort toward equity in digital interventions, there is a significant risk of exacerbating existing health inequalities.

### Strengths and limitations

This review has several strengths. First, this review provides a comprehensive synthesis of 48 studies, including randomised controlled trials, cohort studies, cross-sectional studies, qualitative studies, mixed-methods studies, systematic reviews, and meta-analyses, across five thematic domains, covering a wide range of digital intervention types and study designs.

Second, it integrates quantitative and qualitative evidence, offering a more nuanced understanding of intervention mechanisms. Third, the use of study design specific risk of bias tools (RoB 2, ROBINS-I, AMSTAR 2, NOS, AXIS, CASP, MMAT) enhances methodological transparency.

When analyzing the results of this systematic review, it is important to recognize several inherent limitations (23, 24). Although the search strategy was comprehensive and systematic, selection bias may still exist owing to restriction to English-language publications and no prospective registration of the review protocol. We assessed methodological quality using study-design-specific tools (RoB 2 for randomized controlled trials, ROBINS-I for non-randomized interventional studies, AMSTAR 2 for systematic reviews, NOS for cohort studies, AXIS for cross-sectional studies, CASP for qualitative studies, and MMAT for mixed-methods studies). However, we did not exclude studies based on these assessments, and we did not perform a quantitative meta-analysis. The rapid evolution of technology implies that research published as recently as five years ago may assess tools that differ significantly from today’s digital interventions, which could affect the relevance of the findings, even though the search was confined to the years 2020 to 2026 (2, 7).

Additionally, the evidence is predominantly focused on high-income nations, which restricts its applicability to low- and middle-income contexts (30, 52), and there is a notable lack of evidence for specific groups such as adolescents with disabilities, individuals with chronic illnesses, sexual and gender minorities, and those living in rural areas (4, 35).

The inclusion of children as young as 8 years alongside adolescents aged 10–19 years allows examination of developmental transitions from late childhood through early adolescence. This extended range enabled us to observe how digital intervention effects may differ across adjacent developmental stages, such as the contrasting preferences between younger adolescents (approximately 10–13 years), who responded more readily to playful, sensorimotor activities, and older adolescents (approximately 14–19 years), who preferred goal-setting and self-tracking features.

However, this broad age range also introduces heterogeneity. Developmental differences in cognitive capacity, motivational sources, social needs, and digital technology use patterns within this range may be as substantial as those between adolescents and adults. We addressed this by disaggregating findings by age category where possible (younger group: 10–13 years; older group: 14–19 years) and by explicitly discussing developmental differences throughout the results and discussion sections. Readers should interpret findings with awareness that developmental stage is a critical moderator of intervention effects.

### Implications for design and practice

For those designing interventions, the compiled evidence from quantitative, qualitative, and mixed-methods studies offers several practical guidelines (6, 13, 21). These implications extend to multiple stakeholders, including intervention designers, educators, practitioners, and policy-makers. It is essential to customize developmental stages by aligning features with the cognitive and motivational traits of specific age demographics (15, 21).

It is essential to customize developmental stages by aligning features with the cognitive and motivational traits of specific age demographics (15, 21). Emphasizing relational elements—such as team-based challenges, personalized avatars, private peer groups, and empathetic chatbots—should be viewed as fundamental components that drive engagement rather than mere enhancements (6, 14, 19). Additionally, it is crucial to optimize the timing of digital prompts by synchronizing them with identified activity periods (between 5 and 8 p.m.) and recognizing the difference between situations where digital engagement may hinder physical activity (extended periods of sedentary screen time) and those where it can facilitate it (short interactions during travel or exercise) (26–29).

Proactively tackle equity by creating offline capabilities that include culturally relevant choices, guarantee accessibility on various devices, and take into account programs that provide devices through schools (4, 22, 30). Shift the focus away from merely measuring screen time in both the design and assessment phases, making a clear distinction between how long and how often users engage, and aim for short, contextually relevant interactions instead of extended usage (28, 48, 55). Involve adolescents in every stage of the design process, from brainstorming ideas to testing prototypes (6, 19, 21).

It is essential for educators and practitioners to incorporate digital resources into established frameworks like school curricula and physical education initiatives, rather than treating them as separate entities. Additionally, offering personal support in conjunction with digital elements is crucial, as in-person guidance boosts participation and accountability in ways that solely digital methods cannot achieve (45, 54). To tackle equity challenges, it is important to evaluate access to devices, internet connectivity, and digital skills among the specific communities being targeted (4, 30, 31).

Decision-makers prioritize the inclusion of digital health literacy within educational programs (22, 31). They fund research that is collaboratively designed and emphasizes equity, particularly involving underrepresented communities (30, 53). Additionally, they create regulatory guidelines to guarantee fairness in algorithms, protect data privacy, and ensure transparency in digital health initiatives aimed at young people (13, 49). Furthermore, they promote the enhancement of infrastructure by expanding broadband access and providing digital health resources in schools (22, 30).

### Future research directions

Based on this review, six priority areas for future investigation emerge.

First, progression of development. It is essential to conduct longitudinal research that follows groups from early to late adolescence to understand how reactions to digital interventions evolve with age. Studies should focus on pinpointing particular cognitive and motivational traits associated with various developmental stages and explore whether the elements of interventions need to be modified as adolescents grow older (13, 15, 21, 53).

Second, time-related changes. The observation that MVPA reaches its highest levels from 5 to 8 p.m. (26) needs to be confirmed in various demographic groups. Microrandomized studies are ideal for assessing the causal impacts of the timing of interventions. Additionally, it is important to explore longer-term trends, including seasonal fluctuations and variations between academic sessions and breaks (27, 29, 47).

Third, mechanisms of social interaction and relationships. While elements of relational design (such as team challenges, peer groups, and chatbots) appear to have potential, the exact ways they affect results are not well understood. Subsequent studies ought to investigate mediating factors like perceived social support and self-efficacy, as well as assess if the impacts are consistent among various adolescent groups (6, 14, 17, 19, 21).

Fourth, design with an emphasis on equity. It is essential for research to deliberately incorporate underrepresented groups, utilize collaborative design methods, and assess execution in environments with limited resources. Frameworks from implementation science, such as RE-AIM, can assist in recognizing the contextual elements that affect the uptake and longevity of initiatives within various communities (4, 22, 23, 30, 53).

Fifth, assessment and digital profiling. The shortcomings of using screen time as an indicator of engagement underscore the necessity for more refined measurement techniques. Digital profiling, which integrates passive data gathering (such as accelerometry and application logs) with self-reports triggered by specific events, can enhance insights into how adolescents interact with technology (27, 28, 48, 55).

Sixth, ongoing upkeep and research across different cultures. Extended follow-up periods of 12 to 24 months are essential to assess if early gains are maintained. The existing studies, mainly from affluent nations, restrict the applicability of findings; there is an urgent need for investigations in low- and middle-income environments (4, 11, 30, 45, 46, 52).

## Conclusion

Based primarily on empirical intervention studies (RCTs/quasi-experimental), complemented by mechanistic insights from qualitative research, this review demonstrates that the effectiveness of digital interventions aimed at increasing physical activity among adolescents is not fixed; instead, it significantly hinges on how well these interventions align with three interconnected aspects of adolescent life: developmental suitability, relational relevance, and contextual sensitivity. The ongoing efficacy- engagement gap shows that initial enthusiasm does not guarantee sustained behavioral change. Effective interventions go beyond external incentives by nurturing intrinsic motivation through elements that honor adolescent independence, promote social bonds, and allow for personal identity expression. Features like team challenges, customizable avatars, private peer groups, and empathetic chatbots were associated with a 50% boost in confidence and 120% boost in motivation in one exploratory uncontrolled co-design study, preliminary evidence that requires replication.

Additionally, preliminary evidence from a single study suggests that the timing of activities may play a role, with the likelihood of moderate to vigorous physical activity peaking 13 times higher between 5 and 8 p.m. in that study. The distinction between screen time, which can hinder activity and the frequency of engaging in physical activities, which may encourage it, proves to be critically important, often overlooked in broad screen time analyses. It is essential to prioritize equity from the beginning, as significant gaps in digital health literacy (over 35%), lower engagement rates among racial and ethnic minority youth, and barriers related to device access, connectivity, and neighborhood safety risk exacerbating health inequalities unless there is a conscious effort to align culturally and structurally. Based on available evidence, a promising shift may be moving from a technology-centric approach to one that prioritizes the adolescent perspective. When digital interventions respect the complexities of adolescent identity—acknowledging their developmental, relational, and contextual realities—they can evolve from mere feature lists to rich, identity-affirming, and equitable strategies that weave physical activity into the daily lives of young people. Note: Quantitative estimates here derive from single or limited studies and should be viewed as emerging insights, not established facts.

## Data Availability

The original contributions presented in the study are included in the article/supplementary material, further inquiries can be directed to the corresponding author.

## Appendix A

### Complete search strategy

**Table:**

Database	Search strategy	Number of results
PubMed/ MEDLINE	((adolescent*[Title/Abstract] OR youth[Title/Abstract] OR teen*[Title/Abstract] OR “young people”[Title/Abstract]) AND (“physical activity”[Title/Abstract] OR exercise[Title/Abstract] OR MVPA[Title/Abstract] OR steps[Title/Abstract]) AND (“digital health”[Title/Abstract] OR eHealth[Title/Abstract] OR mHealth[Title/Abstract] OR “mobile app*”[Title/Abstract] OR wearable*[Title/Abstract] OR “fitness tracker*”[Title/Abstract] OR gamif*[Title/Abstract] OR chatbot*[Title/Abstract] OR “artificial intelligence”[Title/Abstract] OR telehealth[Title/Abstract] OR telemedicine[Title/Abstract]))	684
Web of Science	TS = (adolescent* OR youth OR teen* OR “young people”) AND TS = (“physical activity” OR exercise OR MVPA OR steps) AND TS = (“digital health” OR eHealth OR mHealth OR “mobile app*” OR wearable* OR “fitness tracker*” OR gamif* OR chatbot* OR “artificial intelligence” OR telehealth OR telemedicine)	1,086
Scopus	TITLE-ABS-KEY(adolescent* OR youth OR teen* OR “young people”) AND TITLE-ABS-KEY(“physical activity” OR exercise OR MVPA OR steps) AND TITLE-ABS-KEY(“digital health” OR eHealth OR mHealth OR “mobile app*” OR wearable* OR “fitness tracker*” OR gamif* OR chatbot* OR “artificial intelligence” OR telehealth OR telemedicine)	1,044
ScienceDirect	(“adolescents” OR “youth”) AND (“physical activity” OR “exercise”) AND (“digital health” OR “eHealth” OR “mHealth” OR “mobile app” OR “wearable”)	1822
EBSCOhost	AB(adolescent* OR youth OR teen* OR “young people”) AND AB(“physical activity” OR exercise OR MVPA OR steps) AND AB(“digital health” OR eHealth OR mHealth OR “mobile app*” OR wearable* OR “fitness tracker*” OR gamif* OR chatbot* OR “artificial intelligence” OR telehealth OR telemedicine)	1,035
